# A novel chlorhexidine-hexametaphosphate coating for titanium with antibiofilm efficacy and stem cell cytocompatibility

**DOI:** 10.1007/s10856-021-06616-5

**Published:** 2021-11-20

**Authors:** Sarah J. Garner, Mathew J. Dalby, Angela H. Nobbs, Michele E. Barbour

**Affiliations:** 1 Bristol Dental School, University of Bristol, Lower Maudlin Street, Bristol, BS1 2LY UK; 2 Centre for Cell Engineering, Institute of Molecular Cell and Systems Biology, University of Glasgow, Joseph Black Building, University Avenue, Glasgow, G12 8QQ UK

## Abstract

Dental implants are an increasingly popular way to replace missing teeth. Whilst implant survival rates are high, a small number fail soon after placement, with various factors, including bacterial contamination, capable of disrupting osseointegration. This work describes the development of chlorhexidine-hexametaphosphate coatings for titanium that hydrolyse to release the antiseptic agent chlorhexidine. The aim was to develop a coating for titanium that released sufficient chlorhexidine to prevent biofilm formation, whilst simultaneously maintaining cytocompatibility with cells involved in osseointegration. The coatings were characterised with respect to physical properties, after which antibiofilm efficacy was investigated using a multispecies biofilm model, and cytocompatibility determined using human mesenchymal stem cells. The coatings exhibited similar physicochemical properties to some implant surfaces in clinical use, and significantly reduced formation of multispecies biofilm biomass up to 72 h. One coating had superior cytocompatibility, with mesenchymal stem cells able to perform normal functions and commence osteoblastic differentiation, although at a slower rate than those grown on uncoated titanium. With further refinement, these coatings may have application in the prevention of bacterial contamination of dental implants at the time of surgery. This could aid a reduction in rates of early implant failure.

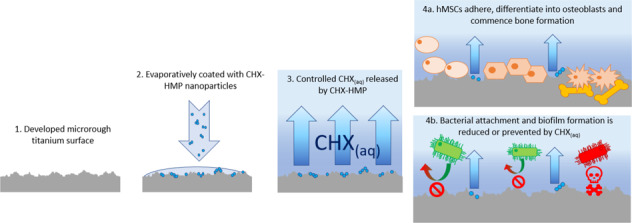

## Introduction

Dental implants are used to rehabilitate patients with congenital or acquired missing teeth. Following surgical placement into alveolar bone, implants are colonised by osteoblasts and undergo osseointegration. Their popularity is continually increasing [1], since they often allow a fixed rather than removable prosthesis and can reduce the need for hard or soft tissue support required by conventional prostheses. However, they are not without problems, one being failure of osseointegration immediately following surgery [2, 3]. Various factors have been associated with this, including peri-operative bacterial contamination of the implant, suboptimal patient response, and iatrogenic issues [4–9].

The oral environment is host to a wide range of microorganisms which form a complex ecosystem, with microbial species interacting with one another as well as with the oral soft and hard tissues. While the presence of microorganisms and of biofilm is inevitable, it is important to manage overall bacterial load as well as the presence and prevalence of particular pathogens to ensure continued good oral health. There is a high chance of peri-operative bacterial contamination from various sources [10]. Bacteria readily adhere to roughened implant surfaces [11, 12] and can form biofilms, which disrupt osseointegration of the implant by mesenchymal stem cells (MSCs). This has been hypothesised as one cause of early implant failures [8]. Whilst these early failures have a lower incidence (1.7–4% of implants; [2, 3]) compared to late failures (1–47%; [13–15]), they render an implant unusable and can result in substantial bone loss, thus limiting or complicating future treatment. Prevention of bacterial contamination at the time of implant placement is therefore desirable, and could contribute to reduced failure rates [16].

Research in the field of implant dentistry to prevent early failures currently centres on maximising the rate and quality of osseointegration and preventing bacterial contamination. The use of antibiotics in prevention of postoperative implant infection is debatable [17, 18], and in a climate of increasing antimicrobial resistance, alternative means to address this should be investigated. Current strategies showing promise in prevention of biofilm formation on implant surfaces are based around three approaches: changes to surface topography to prevent bacterial adhesion, surfaces that release antimicrobial agents for a defined period to prevent adhesion and kill bacteria in the vicinity, and surfaces with permanently bonded antimicrobial agents that prevent attachment of bacteria to the surface in the long term [19, 20].

The aim of this work was to develop a novel coating for a dental implant-like surface using a slow-release compound for the antimicrobial agent chlorhexidine (CHX) in the form of a previously developed hexametaphosphate salt (CHX-HMP) [21, 22]. CHX-HMP is a novel salt of the common antiseptic CHX, which is more commonly encountered as the CHX digluconate or diacetate salts. The HMP salt of CHX has a much lower solubility in aqueous solution than the digluconate or diacetate salts and dissolves slowly on contact with water, thus releasing the CHX gradually over an extended period. A more extensive discussion of CHX-HMP, its preparation and characteristics can be found elsewhere [22]. The rate and magnitude of CHX release from CHX-HMP functionalised materials depends on multiple factors including the local conditions, the doping of CHX-HMP and the matrix in which the CHX-HMP is embedded; the release duration may be days, weeks, months or years [23–26]. In this study, CHX-HMP was further explored as a coating for a titanium substrate and subjected to testing to determine physical characteristics, antibiofilm efficacy and cytocompatibility.

## Materials and methods

### CHX-HMP coatings

Detailed description of the method for production of a sandblasted acid etched (SLA) surface on commercially pure titanium coupons to produce substrates for deposition of CHX-HMP is described in Supplementary Data 1. Briefly, titanium coupons (Ti-Tek (UK) Limited, Birmingham, UK) were sandblasted with 50 µm Al_2_O_3_ particles (Henry Schein® Inc., Melville, USA) followed by a 24 h etch in 2 M sulfuric acid (ThermoFisher Scientific, Loughborough, UK), with an acid change at 16 h (SLA Ti surface) and cleaning with sonication in a soap solution.

A chlorhexidine-hexametaphosphate suspension (CHX-HMP) was precipitated in deionised water (DIW) containing 0.3125% dissolved poloxamer 407 (P407) by addition of aqueous solutions sodium hexametaphosphate and then chlorhexidine digluconate under rapid stirring. This resulted in formation of a white precipitate: CHX-HMP. P407 was used to reduce CHX-HMP particle aggregation. The suspension was diluted 100-fold in DIW and aliquots (100 or 200 µL) were deposited onto SLA Ti coupons and allowed to dry before a washing step in DIW and dry storage (coated substrates denoted CHX-HMP-100 and CHX-HMP-200). A negative control was produced using SLA Ti coupons immersed in stirring DIW for 30 min, denoted SLA Ti henceforth.

### Characterisation of coated SLA Ti

Methods to characterise the CHX-HMP coated SLA Ti are described in Supplementary Data 2: Methods to characterise CHX-HMP coated SLA Ti. This included contact angle and surface roughness measurements, and scanning electron microscopy (SEM) for plain imaging and with energy dispersive X-ray analysis (EDX). Additionally, release of aqueous chlorhexidine (CHX_(aq)_) from CHX-HMP coated substrates was calculated using elution into a simulated tissue fluid (STF, physiologic simulation) or 2 M hydrochloric acid (total available CHX_(aq)_), measurement with spectrophotometry at 255 nm and comparison with known standards.

### Development of biofilm model

To enable testing of the antibiofilm efficacy of the CHX-HMP coatings, a multispecies biofilm model was developed, adapted from that described by Millhouse et al. [27], and organisms selected due to their association with peri-implant disease [10, 28–31]. The bacterial strains used are listed in Table 1. All were grown and maintained under anaerobic conditions (10% hydrogen, 10% carbon dioxide and 80% nitrogen at 45% humidity at 37 °C), with all experimental work carried out under the same, except where explicitly stated.

**Table 1:** Bacterial strains used in this work

Strain ID	Species	Site of isolation	Source
UB2182	*Aggregatibacter actinomycetemcomitans* ATCC 43718 (serotype b)	Subgingival dental plaque	[60]
UB2296	*Fusobacterium nucleatum*	Peri-implantitis lesion	[61]
UB5	*Porphyromonas gingivalis*	Periodontal pocket	Laboratory stock
UB1	*Prevotella nigrescens*	Periodontal pocket	[62]
UB2281	*Streptococcus mitis*	Peri-implantitis lesion	[61]

Detailed description of the selection and maintenance of bacterial strains, and development of the multispecies biofilm model are included in Supplementary Data 3: Bacterial strains, culture conditions and multispecies biofilm model development. Briefly, biofilms were formed at two different inocula (0.5 × 10^7^ or 0.5 × 10^4^ CFU) in nutrient rich media containing 2% v/v sterile-filtered human saliva (TSBYEHM + S) (ethics approval South Central Oxford C Research Ethics, reference 08/H0606/87 + 5).

Initially, *S. mitis* (0.5 mL, 0.5 × 10^7^ or 0.5 × 10^4^ CFU) was added to substrates and incubated under anaerobic conditions for 24 h. After removal of spent medium and gentle washing, *F. nucleatum* was added at the same CFU. The plate was incubated for a further 24 h before removal of spent medium, washing and addition of *A. actinomycetemcomitans, P. gingivalis* and *P. nigrescens* at the same CFU. Coupons were incubated for a further 24 h, resulting in a biofilm grown over 72 h containing five species.

A modification to the model was later introduced to clarify the mechanism by which the CHX-HMP coatings affected biomass formation: this consisted of addition of *S. mitis* at time 0 *and* at 24 h (same CFU), followed by inoculation of *F. nucleatum* at 48 h and *A. actinomycetemcomitans, P. gingivalis* and *P. nigrescens* at 72 h to give a biofilm grown over 96 h.

### Antibiofilm efficacy of CHX-HMP coatings

The antibiofilm efficacy of the CHX-HMP coatings compared to SLA Ti (positive control) was assessed using biomass assays and quantitative PCR (qPCR). Detail of these methods, including qPCR primers used, is contained in Supplementary Data 4: Methods to assess antibiofilm efficacy of CHX-HMP coatings.

### Cytocompatibility testing

Evaluation of the cytocompatibility of the CHX-HMP coatings was conducted in vitro using human mesenchymal stromal cells (hMSCs) (Promocell, Germany). Methods employed were SEM imaging, phosphate staining, immunostaining for tubulin, vinculin, osteopontin and osteocalcin, and metabolomic analysis. Details of methods for each of these, alongside cell culture and maintenance, is included in Supplementary Data 5: Cytocompatibility testing methods.

### Statistical analyses

Statistical analyses for all data (except metabolomics work) were carried out using IBM SPSS Statistics Version 24 (IBM, New York, USA). Data were checked for normality and homogeneity of variance using Shapiro–Wilk and Levene tests. For all tests, *p* values of <0.05 were considered to indicate statistical difference. For normally distributed data with homogeneity of variance, a Student’s *t*-test (2 independent variables) or a 1-way ANOVA (3 or more independent variables) was employed to determine statistical difference, followed by Sidak correction for multiple comparisons, and Tukey’s Honestly Significant Difference (HSD) test used post hoc to identify differences. Where data were normally distributed but did not have homogeneity of variance, a 1-way Welch ANOVA was performed.

Statistical analysis of metabolomics data was performed in Ingenuity Pathway Analysis software (Qiagen) and Metaboanalyst software and consisted of calculation of a *z*-score to indicate a predicted pathway activation or deactivation (*z*-scores > or <2 respectively) with Fisher’s Exact where indicated, and 1-way ANOVA or Kruskal Wallis followed by Fisher’s LSD post hoc in Metaboanalyst. Principal Component Analysis (PCA) plots were also performed for each surface for various metabolomic functions. Significance was assumed for all test results of *p* < 0.05.

## Results

### CHX-HMP coatings

The mean contact angle and surface roughness (as *R*_a_) for SLA Ti, CHX-HMP-100 and CHX-HMP-200 surfaces are shown in Table 2. Statistical analysis demonstrated no difference in contact angle between SLA Ti and CHX-HMP coated samples. All surfaces exhibited a slight hydrophilic tendency (<90˚ contact angle). There were no statistical differences between *R*_a_ values measured in the *X* and *Y* directions for all coatings indicating surfaces were isotropic (Table 2). There were no statistical differences in *R*_a_ value between SLA Ti and CHX-HMP-100 (*p* = 0.279) or CHX-HMP-200 (*p* = 0.068) coating (Table 2). However, CHX-HMP-100 and CHX-HMP-200 were statistically different from one another (*p* = 0.01; all one way ANOVA and Tukey HSD post hoc; *n* = 8).

**Table 2:** Physical and chemical characteristics of SLA Ti and CHX-HMP coatings

	SLA Ti	CHX-HMP-100	CHX-HMP-200
Mean contact angle (SD)	84˚ (7.28˚)	82˚ (5.95)	82˚ (6.58)
Mean surface roughness—*R*_a_ (SD) [*P*-value for *R*_a_ in *X* and *Y* directions on surface (Student’s *t* test)]	1.25 µm (0.06 µm) [0.214]	1.31 µm (0.08 µm) [0.356]	1.16 µm* (0.04 µm) [0.098]
Chlorine peaks present in EDX	Absent	K_α_1 and K_α_2	K_α_1 and K_α_2
Phosphorus peaks present in EDX	Absent	K_α_1 and K_α_2	K_α_1 and K_α_2

*SD* standard deviation

*p = 0.01 relative to CHX-HMP-100, as determined by one way ANOVA and Tukey HSD post hoc (*n* = 8)

**Fig. 1:**
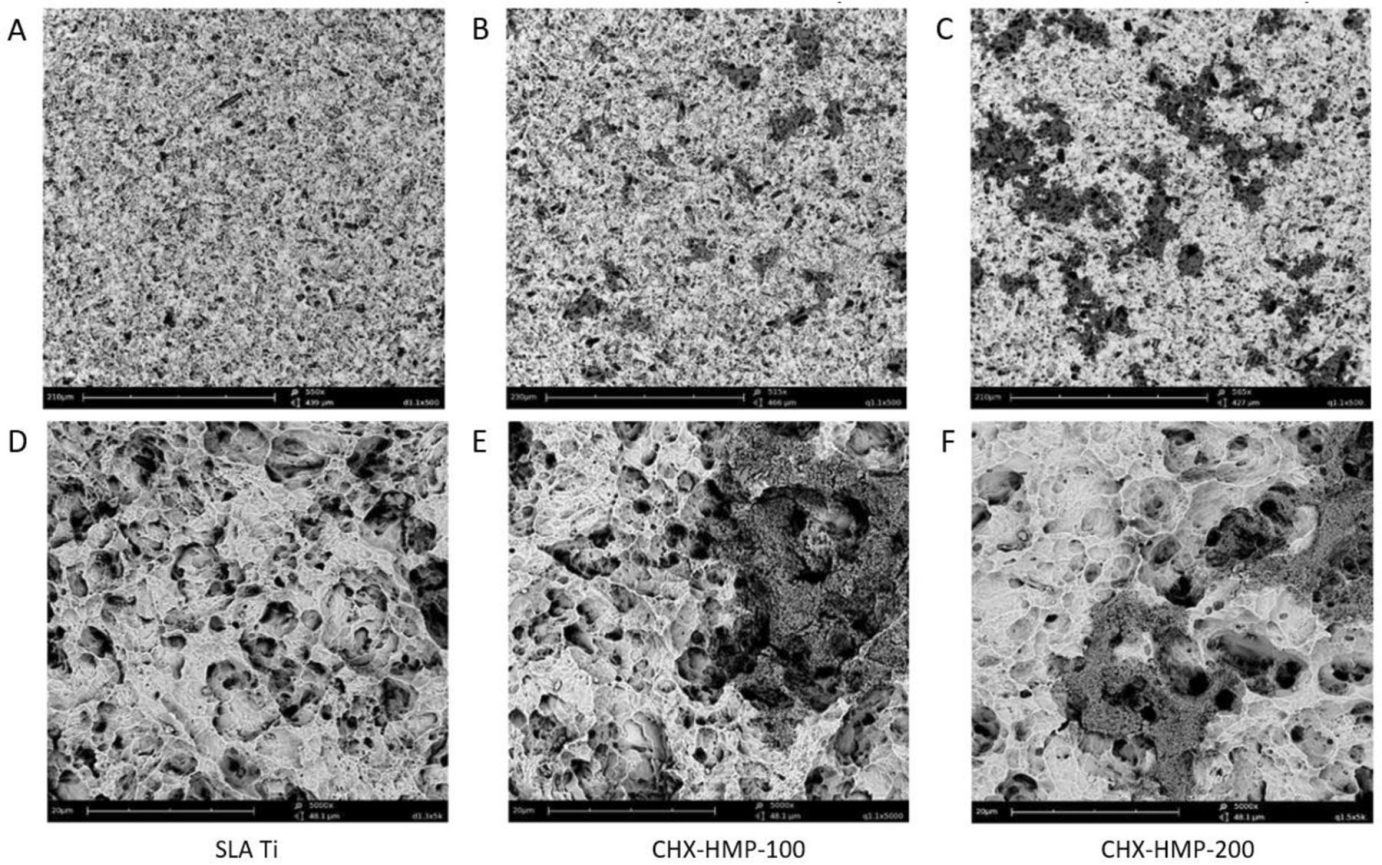
Representative SEM micrographs of **A**, **D** SLA Ti, **B**, **E** CHX-HMP-100, and **C**, **F** CHX-HMP-200 coatings showing number and distribution of CHX-HMP deposits. Scale bars 210 µm (**A**–**C**) and 20 µm (**D**–**F**)

SEM imaging demonstrated the surface morphology of the developed SLA Ti surface, and the discontinuous deposition of CHX-HMP deposits on the two different coatings (Fig. 1). Composition of the deposits was confirmed using SEM-EDX analysis (Table 2), including presence of chlorine and phosphorous, which are present in the CHX-HMP structure [32]. A greater number of larger deposits were seen on the CHX-HMP-200 coating.

**Fig. 2:**
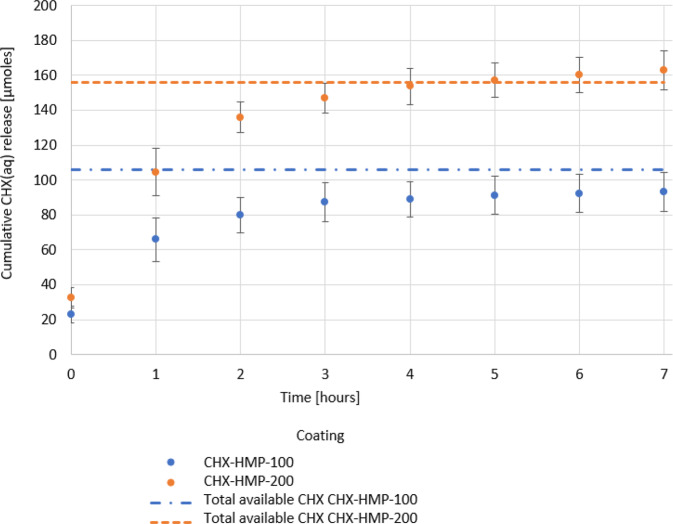
Mean cumulative CHX_(aq)_ release per metre squared of CHX-HMP coated SLA Ti. Elution into STF at room temperature. Error bars show standard deviation. Dotted lines represent pooled mean total available CHX on each coating type as determined using elution into 2 M HCl (10 specimens per coating type)

Elution studies in STF showed most of the CHX_(aq)_ release from CHX-HMP coated substrates occurred over the first 7 h (Fig. 2). A separate 14 day elution demonstrated no further substantial CHX_(aq)_ release (Supplementary Data 6: CHX_(aq)_ elution into STF over 14 days). In the presence of 2 M HCl, all available CHX_(aq)_ was immediately released from both CHX-HMP coatings (Fig. 2). The mean total available CHX_(aq)_ released from the CHX-HMP-100 and CHX-HMP-200 coatings was 106 and 156 µmoles/m^2^, respectively, resulting in equivalent concentrations of 0.001% (21.2 µM) and 0.0015% (31.2 µM).

### Antibiofilm efficacy of CHX-HMP coatings

**Fig. 3:**
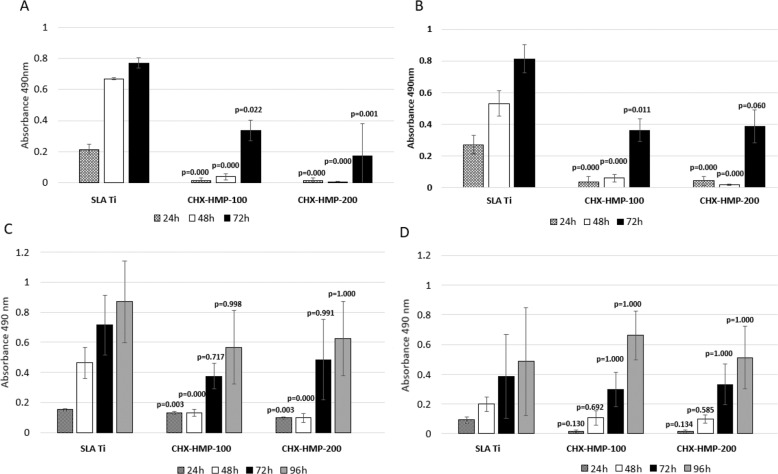
Biomass of biofilms grown on uncoated and CHX-HMP coated SLA Ti. Biofilms were formed using bacterial inocula of 0.5 × 10^4^ CFU (**A**, **C**) or 0.5 × 10^7^ CFU (**B**, **D**), and grown for 72 h (**A**, **B**) or 96 h (**C**, **D**), the latter including a re-inoculation step with *S. mitis* after 24 h. Biomass was quantified by safranin stain and acetic acid release. Error bars represent standard deviations. *P*-values are shown relative to SLA Ti surface as determined by 1-way ANOVA with Sidak correction post hoc (*n* = 3)

A 5-species biofilm model was used to determine antimicrobial efficacy of the CHX-HMP coatings. A steady increase in biofilm biomass formation over 72 h was seen on SLA Ti. By contrast, less biomass formed on both CHX-HMP coatings at both 0.5 × 10^4^ and 0.5 × 10^7^ CFU inocula (Fig. 3A, B). A 96 h biofilm model was also employed to clarify the mechanism by which the CHX-HMP coatings affected biomass formation; by adding a second inoculation of *S. mitis* at 24 h, it was possible to ascertain the antibiofilm effect of the CHX-HMP coatings persisted beyond 24 h and a medium change. Results of this model showed less biomass was found on the CHX-HMP coated substrates at 24 and 48 h at the 0.5 × 10^4^ inoculum compared to SLA Ti control (Fig. 3C). At the higher 0.5 × 10^7^ inoculum, however, there were no significant differences in biomass between SLA Ti and the CHX-HMP coatings at any time point (Fig. 3D).

Table 3 shows the results of qPCR analysis of biofilm composition after 72 h on control and test substrates. There were no statistical differences to biofilm composition, regardless of presence or absence of CHX-HMP. *S. mitis* was the predominant organism, constituting 95.6–99.9% of the biofilms formed. The remaining four species each made up less than 1% of the biofilm in most instances.

**Table 3:** Mean percentage composition of 72-h multispecies biofilms formed on SLA Ti and CHX-HMP coatings

	Mean % of each species within total biofilm^a^				
Coating and inoculum	*S. mitis*	*F. nucleatum*	*A. actinomycetemcomitans*	*P. gingivalis*	*P. nigrescens*
SLA Ti: 0.5 × 10^4^ inoculum	99.93 (0.12)	0.06 (0.12)	0.00 (0.00)	0.00 (0.00)	0.00 (0.00)
SLA Ti: 0.5 × 10^7^ inoculum	98.41 (1.41)	0.95 (0.96)	0.09 (0.10)	0.02 (0.02)	0.53 (0.49)
CHX-HMP-100: 0.5 × 10^4^ inoculum	98.26 (2.34)	0.53 (0.54)	0.23 (0.45)	0.00 (0.00)	0.98 (1.96)
CHX-HMP-100: 0.5 × 10^7^ inoculum	98.82 (0.41)	0.49 (0.27)	0.09 (0.06)	0.04 (0.02)	0.56 (0.53)
CHX-HMP-200: 0.5 × 10^4^ inoculum	98.73 (1.25)	1.47 (1.86)	0.24 (0.35)	0.01 (0.02)	0.38 (0.67)
CHX-HMP-200: 0.5 × 10^7^ inoculum	95.57 (3.84)	0.67 (0.73)	2.51 (4.57)	0.04 (0.05)	1.20 (1.62)

^a^Percentage composition by gDNA mass. Figures in brackets indicate standard deviation. No significant differences between coating type or inocula for any species, as determined by 1-way ANOVA with Sidak post hoc (*n* = 4)

### Cytocompatibility testing

Cytocompatibility testing was performed over 1–28 days to determine hMSC response to the CHX-HMP coatings with respect to survival, attachment, growth, proliferation, metabolism and differentiation. This facilitated a more complete understanding of the mechanisms by which released CHX_(aq)_ exerted cytotoxic effects, and the likely sequalae of these, allowing assessment of the potential suitability of the CHX-HMP coatings for use on dental implants.

**Fig. 4:**
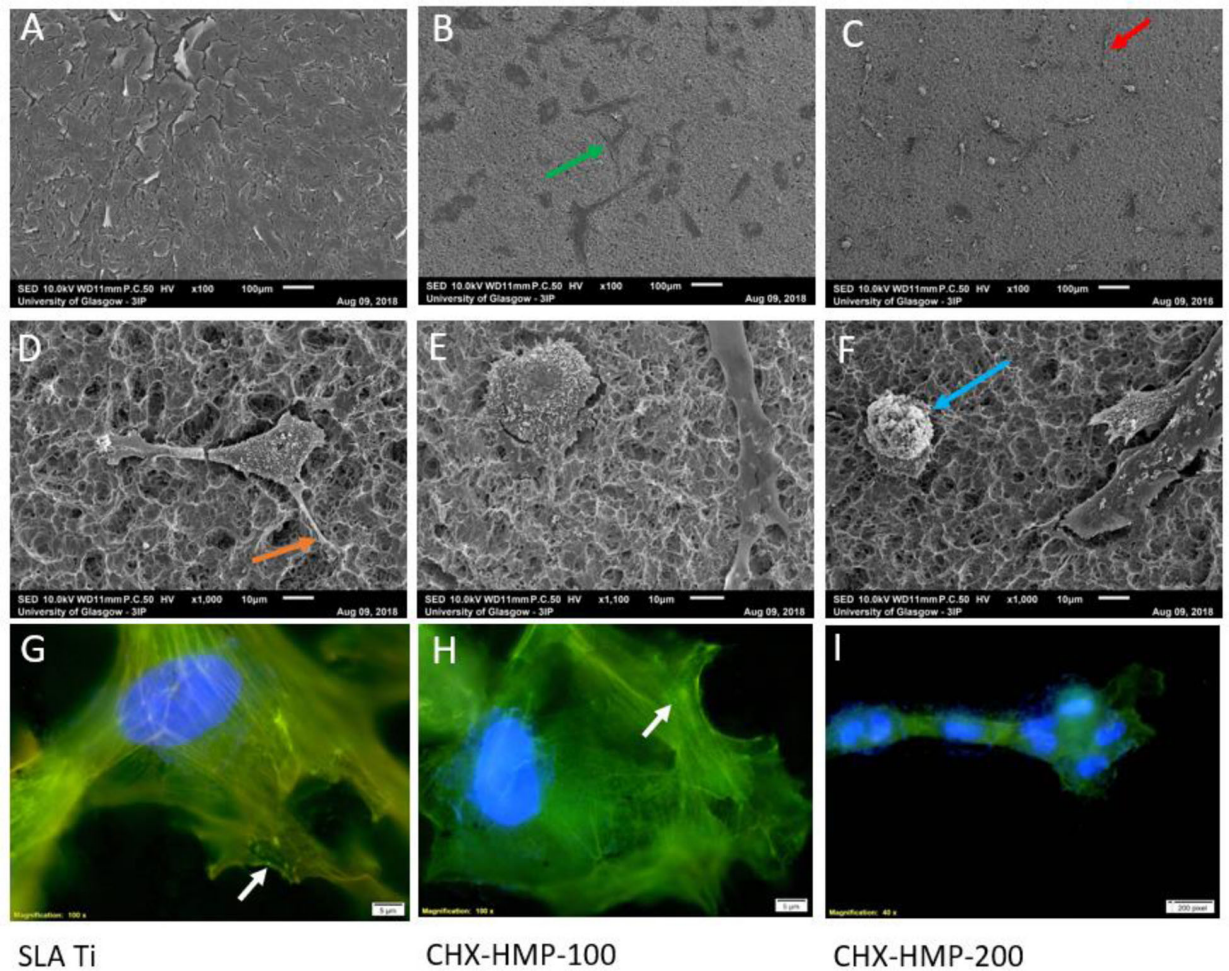
Adhesion of hMSCs to SLA Ti and CHX-HMP coatings after 7 days’ growth. SEM images (**A**–**F**) show different morphologies. Arrows: red—spindle shaped cells; green—stellate morphology; blue—spherical morphology; orange—cell processes interacting with surface. Colour composite micrographs of vinculin immunostaining (**G**–**I**) show vinculin deposits (white arrows) within focal adhesions (green) and DAPI-stained nuclei (blue). Scale bars: **A**–**C**, 100 µm; **D**–**F**, 10 µm; **G**–**I**, 5 µm

SEM imaging demonstrated ready adherence and growth of hMSCs on SLA Ti (Fig. 4A, D). On the CHX-HMP-100 coating, cells were fewer, smaller and more spaced, some with a stellate morphology, whilst others were spherical (Fig. 4B, E). On the CHX-HMP-200 coating, fewer cells were present still, with a larger number exhibiting a spherical, unspread morphology (Fig. 4C, F). Vinculin immunostaining demonstrated larger, more frequent focal adhesions in hMSCs grown on SLA Ti compared to either of the CHX-HMP coatings. The reduction in adhesion was most notable for hMSCs on the CHX-HMP-200 coating (Fig. 4G–I).

**Fig. 5:**
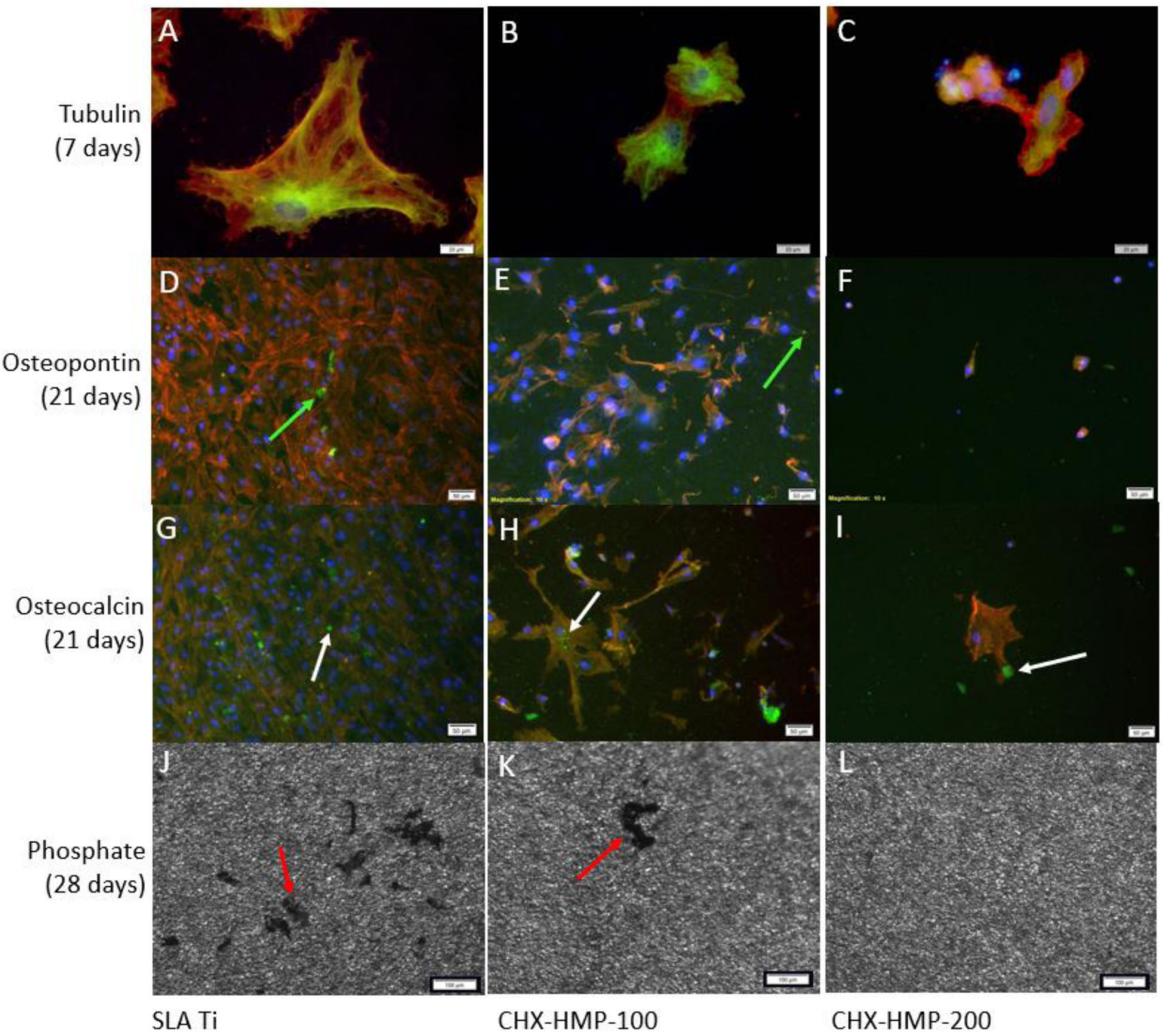
Assessment of hMSC metabolism and differentiation after growth on SLA Ti, CHX-HMP-100, and CHX-HMP-200 coatings. Images **A**–**C** are colour composite micrographs of hMSCs grown for 7 days showing tubulin staining of microtubule networks (green), nuclei (blue), and actin (red). Images **D**–**F** are colour composite micrographs of hMSCs grown for 21 days showing osteocalcin staining of calcified extracellular deposits (green arrows to green deposits), nuclei (blue), and actin (red). Images **G**–**I** are colour composite micrographs of hMSCs grown for 21 days showing osteopontin staining of calcified extracellular deposits (white arrows to green deposits), nuclei (blue), and actin (red). Images **J**–**L** are light micrographs showing silver stained deposits of phosphate (red arrows) produced by hMSCs grown for 28 days. Scale bars: **A**–**C** 20 µm, **D**–**I** 50 µm, **J**–**L** 100 µm

Tubulin immunostaining demonstrated large, active tubulin networks on hMSCs grown on SLA Ti (Fig. 5A). hMSC cells and their associated tubulin networks were smaller and less organised on the CHX-HMP-100 coating and showed no organisation for cells on the CHX-HMP-200 coating (Fig. 5B–C), with close spacing of DAPI-stained nuclei suggestive of diminutive cells growing in close association or on top of each other.

After 21 days’ growth, composite micrographs showed a confluent layer of hMSCs present on SLA Ti, with numerous deposits of green coloured osteocalcin and osteopontin visible throughout (Fig. 5D, G). Cells were far less frequent and more spaced out on the CHX-HMP-100 coating, and fewer deposits of osteocalcin and osteopontin were present (Fig. 5E, H). Very few cells were present on the CHX-HMP-200 coatings, which were smaller and with altered morphology compared to SLA Ti and CHX-HMP-100, with no osteocalcin deposits and only very infrequent deposits of osteopontin (Fig. 5F, I).

After 28 days, phosphate deposits were seen on both SLA Ti and CHX-HMP-100, although the latter had fewer, whilst none were detected on CHX-HMP-200 (Fig. 5J-L).

**Fig. 6:**
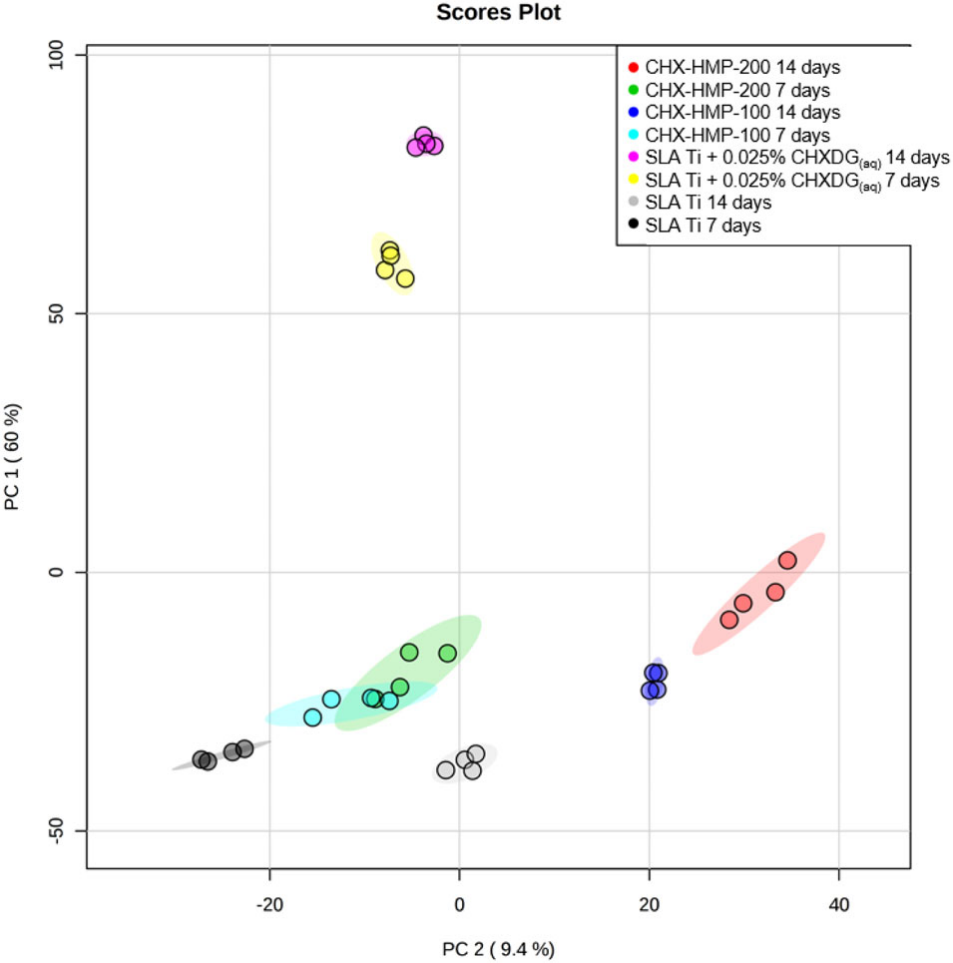
Combined PCA plot for data on all surface types at both 7 and 14 days. Data has been log transformed to facilitate presentation, after normalisation to SLA Ti at 7 days (*n* = 1). Filled circles represent single specimens, shaded area represents region of 95% confidence interval. Key: CHX-HMP-200 14 days—red, 7 days—green; CHX-HMP-100 14 days—dark blue, 7 days—light blue; SLA Ti + 0.025% CHXDG_(aq)_ 14 days—pink, 7 days—yellow; SLA Ti 14 days—grey, 7 days—black. Cell responses were most different on SLA Ti + 0.025% CHXDG_(aq)_ (pink and yellow) at both time points

Metabolomic analysis was undertaken on cells grown on SLA Ti+/− CHX-HMP coatings at 7 and 14 days. A PCA plot indicated that, in comparison to SLA Ti, hMSC responses were most different on the CHX_(aq)_ control (Fig. 6 pink and yellow) at both time points. Responses of hMSCs on both CHX-HMP coated surfaces were similar to control at day 7 but diverged more by 14 days of culture (Fig. 6).

From the same data, networks were produced for key cell pathways, and indicated significant activations or deactivations of relevant functions on CHX-HMP coatings compared to SLA Ti. Examples of these are presented in Supplementary Data 10.

A summary of key relevant predicted cell function changes (compared to SLA Ti at the corresponding time point) at 7 and 14 days formed from these networks, and metabolomic data analysis and computation of *z*-scores for each surface type, is given in Table 4. All statistically data from this analysis are shown in Supplementary Data 9.

**Table 4:** Summary of significant changes to relevant hMSC functions on test surfaces at 7 and 14 days relative to SLA Ti

Function/Network changes	CHX-HMP-100		CHX-HMP-200		SLA Ti + 0.025% CHXDG_(aq)_	
	7 days	14 days	7 days	14 days	7 days	14 days
ALP activation	Activated	Deactivated	Activated	Deactivated	Activated	Deactivated
Apoptosis	Activated	Deactivated	Activated	Activated	Activated	Activated
Ca^2+^ mobilisation	NS	NS	NS	NS	Activated	Deactivated
Mitochondrial dysfunction	Activated	Deactivated	Activated	NS	NS	NS
Necrosis	Activated	Deactivated	Activated	Activated	Activated	Activated
Generation of ROS	Activated	NS	Activated	Deactivated	NS	Activated

Statistically significant changes to cell functions on each test surface compared to SLA Ti at same time point are shown. NS—no significant difference compared to SLA Ti control at same time point. Results are predicted changes to functional networks, based on *z*-scores from metabolomic analysis (Supplementary Data 9)

## Discussion

A SLA type surface finish for titanium was selected for use in this work, since this is one of the most commonly used preparations for dental implants [33]. A technique for reliable production of this type of finish was devised that resulted in a surface with similar characteristics to those found on SLA type implant surfaces in research and clinical use [34, 35].

Characterisation by means of contact angle measurement and *R*_a_ indicated favourable surface properties: hydrophilicity of implant surfaces has been shown to increase adhesion of plasma proteins and favour blood clot retention [1, 36]. Ultimately this results in higher bone to implant contact [37], favouring improved osseointegration [35, 38–40]. A moderately rough surface, as developed here [41], is also considered to favour osseointegration due to increased attachment of the blood clot and, later, of osteoblast precursor cells and thus bone ingrowth [1, 33, 40, 42, 43]. Whilst many microscopically rough surfaces allow formation of undesirable bacterial biofilms [11, 12], an *R*_a_ of 1–2 µm is considered to give the best compromise with regards to facilitating adhesion of hMSCs and ultimately osseointegration [41]. As such, the physical surface characteristics for the SLA Ti surfaces developed here could be expected to facilitate osseointegration.

CHX_(aq)_ elution kinetics into a physiological medium demonstrated that almost all CHX_(aq)_ release from the CHX-HMP coatings occurred within the first 7 h, and reached levels that exceeded the MIC for CHXDG_(aq)_ determined for each test bacterium (Supplementary Data 8: MIC and MBC to CHXDG_(aq)_). As a consequence, however, the majority of CHX_(aq)_ was removed at the first medium change (24 h) in the biofilm model. Nonetheless, the short-term inhibitory effects of CHX_(aq)_ on primary coloniser *S. mitis* still impacted subsequent biofilm formation, and the extent of these effects correlated with microbial burden. These findings are in accordance with the accepted biofilm model [44] in which adhesion of primary colonisers such as *S.mitis* are critical to subsequent attachment of bridging organisms and secondary colonisers: here, when the attachment of *S. mitis* was prevented, subsequent biomass accretion was significantly reduced.

When *S. mitis* was reinoculated at 24 h in the 96 h model, thus presumably negating the effect of released CHX_(aq)_ which had been removed during the medium change, the reduction in biomass on both CHX-HMP coatings persisted only as far as 48 h at the lower inoculum. There was no significant difference in biomass seen at any timepoint on any surface for the higher inoculum in the 96 h model. These findings indicate that both duration of CHX_(aq)_ presence in the medium and microbial burden were important factors in the effect of the CHX-HMP coatings on biofilm growth, and that the effect on *S. mitis* of CHX_(aq)_ released from these coatings did not persist beyond 24 h.

A similar elution profile and antibiofilm effect has been observed previously with nanoparticle encapsulated CHX_(aq)_. Seneviratne et al. [45] showed that whilst active CHX_(aq)_ release only occurred during the first 6 h, this was adequate to suppress growth of a similar four-species anaerobic biofilm for up to 72 h.

There were no significant differences in the composition of the biofilms formed here on SLA Ti compared to CHX-HMP coated surfaces, with the primary coloniser *S. mitis* predominating. This is comparable to studies using similar models, with early coloniser species prevailing in multispecies, anaerobic biofilms [27, 46]. *Streptococcus* species are known to predominate in periodontal and peri-implant health [28, 47, 48], whilst in disease, their proportion is reduced [10]. It is therefore encouraging that the CHX-HMP coatings did not alter the composition of the bacterial biofilm to a more pathogenic one. Since implants are ideally placed into a healthy oral environment, the model used here should mimic the conditions under which an implant surface might become contaminated during placement. Therefore, these data suggest that the CHX_(aq)_ release profile of the CHX-HMP coatings has potential to prevent biofilm formation by bacterial species relevant to implant infection.

The CHX_(aq)_ release duration of these experimental coatings is nevertheless considerably shorter than those previously reported using the same CHX-HMP formulation [23–25] which have ranged from weeks to months. Here, CHX-HMP was deposited onto SLA Ti solely by evaporation, whereas in other studies, it was incorporated in substantial volumes into absorbent materials, either by prolonged immersion, solubilisation of the substrate or as a component of a dual-mix material. On SLA Ti, which has no known absorbent properties, it is unlikely that any CHX-HMP was physically retained within the material and so therefore, the small volume of ultimately soluble CHX-HMP which was deposited, hydrolysed rapidly on exposure to a dilute aqueous solution. However, as shown above, this modest release profile was adequate to suppress biomass accretion by inhibition of primary coloniser attachment and proliferation, most likely because the MIC for *S. mitis* was exceeded by the CHX_(aq)_ released from the CHX-HMP coatings.

Regarding potential future clinical application, cytocompatibility of the SLA Ti surface and CHX-HMP coating was an important consideration. SEM imaging demonstrated attachment to and proliferation of hMSCs on SLA Ti, with an abundance of large cells exhibiting a spread morphology. The presence of bright, dense tubulin networks indicated active intracellular transport and thus low levels of cell stress on this surface [49], whilst numerous focal adhesions suggested that SLA Ti would later stimulate hMSC osteogenic differentiation via activation of ERK1/2 and phosphorylation of RUNX2 [50]. This was further supported by extensive osteocalcin and osteopontin immunostaining and the presence of calcium phosphate at 28 days on SLA Ti, confirming late-stage osteoblastic differentiation. Such observations are in agreement with other titanium surfaces with similar *R*_a_ values [42, 51].

SEM, vinculin and tubulin staining on cells grown on CHX-HMP coatings in this work demonstrated reduced attachment of hMSCs, smaller cells with altered morphology, reduced numbers of focal adhesions and evidence of altered metabolism, similar to changes seen in osteoblast-like cells exposed to CHX_(aq)_ in other studies [52–54]. Vinculin is a membrane protein associated with cell adhesion to a surface, and is involved in linkage of integrin adhesion molecules to the actin cytoskeleton. The presence and size of labelled vinculin gives qualitative information on cell adhesion to a surface. When integrins interact with the extracellular matrix, phosphorylation of Rho-associated protein kinase (ROCK) and myosin light chain kinase (MLCK) occurs, causing contraction of the cell cytoskeleton, and groups of integrins form cell adhesions containing vinculin. Formation of these adhesions causes an intracellular signalling cascade and these cytoskeletal signalling events have been shown to play a key role in deciding MSC fate. Tubulin is a component of the cytoskeleton, and the metabolism of the cell can be inferred from a tubulin immunostain. Microtubules are involved in multiple transport pathways within the cell thus, the density of the tubulin networks qualitatively indicates more or less cell activity. A rounded cell morphology was suggestive of apoptosis or irreversible cell damage, attributable to the presence of CHX_(aq)_ [52, 55], and a dose-response effect to CHX-HMP was apparent, similar to that observed with CHX_(aq)_ [52, 56]. These findings imply that the CHX-HMP coatings assessed here disrupted cells by a similar mechanism to that reported previously.

CHX_(aq)_ exerts cytotoxic effects via multiple mechanisms including reduction in mitochondrial membrane potential, ATP depletion, increased cytoplasmic Ca^2+^ concentration and increased generation of reactive oxygen species (ROS), resulting in apoptosis and necrosis [52, 53, 55, 57, 58]. The severity of these effects is dependent on exposure duration, concentration, CHX_(aq)_ release rate and the presence of cytoprotective factors such as FBS [52, 53, 57, 58].

Metabolomic data collected from hMSCs grown for 7 days on CHX-HMP coatings predicted cytotoxic effects, with activation of apoptosis and necrosis signalling, increased ROS production and increased mitochondrial dysfunction, indicative of cell stress, as has been observed previously [52, 57, 59]. By 14 days after the CHX_(aq)_ present in the environment had been removed through media changes, many of these effects had been reversed, or were no longer significantly different to SLA Ti. Whilst hMSC responses on CHX-HMP coatings were different to those on SLA Ti, they were more similar than those seen with the CHX_(aq)_ control. This suggests that the slower CHX_(aq)_ release from the CHX-HMP coatings may induce fewer adverse effects on cell metabolism than the CHX_(aq)_ control, which showed increased ROS generation, mitochondrial permeabilisation and apoptosis.

Whilst the metabolomic effects appeared to be transient, reduced or minimal osteocalcin, osteopontin and phosphate deposits within hMSCs on the CHX-HMP coatings relative to SLA Ti persisted. Interestingly, Kotsakis et al. [53] reported no effect on osteocalcin activity at 3 and 5 days in murine osteoblasts following a 20-second exposure to a much higher concentration of CHX_(aq)_ (0.0672%) than that released by the CHX-HMP coatings in this work over a number of hours. This again shows that CHX_(aq)_ toxicity to hMSCs is a function of concentration and exposure duration. Both of these parameters would need to be considered when further developing the CHX-HMP coating.

## Conclusion

Maximising adherence of hMSCs whilst minimising that of microorganisms is a key challenge of implant surface development [16, 20]. The data presented here indicate that the CHX-HMP-100 coating on SLA Ti had the capability to reduce biofilm formation by relevant oral microorganisms, and simultaneously allowed adhesion, growth and proliferation of hMSCs. CHX-HMP-100 appeared better tolerated by the hMSCs than CHX-HMP-200. Whilst CHX-HMP-100 did induce adverse effects, network predictions from metabolomic analyses suggested that, if hMSCs were able to survive the initial CHX_(aq)_ insult, they were able to recover some function and continue to proliferate and even commence osteoblastic differentiation once the CHX_(aq)_ was removed. As such, hMSCs may be able to tolerate the CHX-HMP-100 coating. Given its antibiofilm effects, this coating could merit further investigation and optimisation as a potential implant coating with the aim of addressing early dental implant infection and failure.

## Supplementary information

Supplementary Data

## Data Availability

Data freely available from this link: data.bris Data from Garner et al 2020 (06-2020).
