# Supplementary material for: A novel chlorhexidine-hexametaphosphate coating for titanium with antibiofilm efficacy and stem cell cytocompatibility
Source: J Mater Sci Mater Med. 2021 Nov 20;32(12):139. doi: 10.1007/s10856-021-06616-5 (PMC8605967; doi:10.1007/s10856-021-06616-5)
Supplement: Supplementary file 1 — Supplementary Data [file 10856_2021_6616_MOESM1_ESM.docx]

# Supplemental Data

## Supplemental Data 1: Method for production of SLA Ti and CHX-HMP coatings

A roughened surface was produced on commercially pure titanium coupons (Ti-Tek (UK) Limited, Birmingham, UK) by sandblasting with 50 µm Al_2_O_3_ particles (Henry Schein® Inc., Melville, USA) at 3 MPa followed by a 24 hour etch in 2 M sulfuric acid (ThermoFisher Scientific, Loughborough, UK), with an acid change at 16 hours (SLA Ti surface). Coupons were subsequently cleaned by sonication in a deionised water (DIW) and soap solution followed by DIW, and stored dry at room temperature.

To precipitate the CHX-HMP, 0.25 g of poloxamer 407 (P407; Sigma Life Science, Sigma-Aldrich Company Ltd. Dorset, UK) was dissolved in 80 mL DIW at room temperature and pressure at a constant stirring rate. The function of the P407 was to reduce CHX-HMP aggregation, which had been observed previously [21]. Next, 5 mL of 100 mM sodium hexametaphosphate (Sigma) was added, followed by 15 mL 100 mM chlorhexidine digluconate (Sigma). This resulted in formation of a white precipitate, denoted CHX-HMP. This suspension was diluted 100-fold in 2 steps with DIW, then 100 or 200 µL aliquots were deposited onto the SLA Ti coupons in a laminar flow hood and allowed to evaporate dry. The samples were washed in stirring DIW for 1 minute to remove excess reagents and loosely attached CHX-HMP deposits, before air drying. Coated coupons were stored dry and covered at room temperature. Henceforth the 100 or 200 µL CHX-HMP coatings are denoted CHX-HMP-100 and CHX-HMP-200, respectively. To produce a negative control, SLA Ti coupons were immersed in 100 mL stirring DIW for 30 minutes followed by the same washing and drying step as previous. These control samples are denoted SLA Ti.

## Supplemental data 2: Methods to characterise CHX-HMP coated SLA Ti

Contact angle for SLA Ti and CHX-HMP-100 and CHX-HMP-200 coatings was determined using a Drop Shape Analyzer DSA100 and Advance Software (Kruss GmbH, Hamburg, Germany). A measurement was taken using a 2 µL droplet DIW, sessile drop method, at 25°C. Associated software was used with the Ellipse-Tangent fitting method to calculate contact angle 2 seconds after water droplet deposition. Left and right contact angles were measured and a mean calculated from 4 measurements per substrate (n=10), with complete air drying between measurements.

Surface roughness was determined as R_a_ using non-contact optical profilometry (Proscan 2100 profilometer, CHR-150-L Chromatic confocal sensor S11/03; Scantron Ltd, Taunton, UK) with vertical range 300 µm and z resolution of 12 nm. Proscan 2000 software (Scantron Ltd) was used for data analysis. Scan rate was 100 Hz, step size 0.01 mm, and total scan area 1.5 mm^2^. A surface filter of 40 was applied to remove surface waviness. R_a_ was calculated as a mean of X and Y readings in 4 different areas per substrate (n=8).

SLA Ti +/- CHX-HMP coating were subjected to SEM imaging on a Phenom Pro (Phenom-World, Eindhoven, Netherlands) at 10 kV. Specimens were sputter coated with gold/palladium alloy (Emitech SC7620, Quorum Technologies Ltd, Laughton, UK). SEM EDX analysis was carried out on a JEOL JSM-IT300 SEM (JEOL (UK) Ltd, Welwyn Garden City, UK) at accelerating voltage 15 kV, working distance 10 mm and pixel dwell time of 250 µs with an X-Max X-ray detector, using Aztec software (Oxford Instruments, Abingdon, UK).

Release of CHX_(aq)_ from SLA Ti +/- CHX-HMP coating was determined by elution. Substrates were placed into disposable polystyrene semi-micro cuvettes (Brand Gmbh + Co KG, Wertheim, Germany) and filled with 2.5 mL of solution (see below) to allow elution of CHX_(aq)_ from the CHX-HMP coating following its hydrolysis. Cuvettes were sealed with Parafilm®(Sigma) to prevent evaporation of liquid. Cuvettes were placed on a rotating platform (SSM1, Stuart Scientific, Stone, UK) at 150 rpm at 20°C. CHX_(aq)_ release was determined as cumulative release per unit area of coating using absorbance at 255 nm (Biochrom Libra S60; Biochrom Ltd, Cambourne, UK). Absorbance readings at 255 nm for uncoated substrates were used to determine background-subtracted CHX_(aq)_ release values. Elution kinetics were determined in a simulated tissue fluid (STF), based on one reported previously [62]. The following reagents were dissolved in stirring DIW at 20˚C in turn: NaCl 3.998 g/L, NaHCO_3_ 0.175 g/L, KCl 0.112 g/L, K_2_HPO_4_.3H_2_O 0.114g/L, MgCl_2_.6H_2_O 0.153 g/L, 1 M HCl 20 mL, CaCl_2_ 0.139 g/L, Na_2_SO_4_ 0.036 g/L, (CH_2_OH)_3_CNH_2_ 3.029 g/L. The pH was adjusted to 7.4 with 1 M HCl before storage at 4˚C until use. To determine total available CHX**_(aq)_** on substrates, specimens were immersed in 1.5 mL 2 M HCl, resulting in rapid solubilisation of CHX-HMP and consequent release of all CHX**_(aq)_**, which was quantified as described previously using spectrophotometry at 255 nm.

## Supplemental data 3: Bacterial strains, culture conditions and multispecies biofilm model development

*Fusobacterium nucleatum, Porphyromonas gingivalis,* and *Prevotella nigrescens* were maintained on Fastidious Anaerobic Agar (LAB M limited, Heywood, UK) supplemented with 5% defibrinated horse blood (TCS Biosciences Ltd, Buckingham, UK), while *Aggregatibacter actinomycetemcomitans* was maintained on Tryptone Soy Agar (TSA) (Oxoid Limited, Basingstoke, UK) supplemented with 0.1% yeast extract (YE) (Becton Dickinson, Oxford, UK). All species were grown under anaerobic conditions: 10% hydrogen, 10% carbon dioxide and 80% nitrogen at 45% humidity at 37˚C (Whitley A85 TG anaerobic workstation, Don Whitley Scientific Ltd, Shipley, UK). *Streptococcus mitis* was maintained on TSA supplemented with 5% defibrinated horse blood and grown at 37˚C in an oxygen-depleted atmosphere ‘candle jar’. For broth cultures, all species were grown in Tryptic Soy Broth (Oxoid Limited, Basingstoke, UK) supplemented with 0.1% (w/v) YE, 0.1% (v/v) hemin (Sigma Life Science, Sigma-Aldrich Company Ltd. Dorset, England) and 0.5% (v/v) menadione (Sigma Life Science) (TSBYEHM). Cultures were incubated for 24-48 hours anaerobically (16 hours in a candle jar for *S. mitis*) until bacterial growth was visible.

### Multispecies biofilm formation

The multispecies biofilm model was adapted from Millhouse *et al.* [27], and organisms selected due to their association with peri-implant disease [10], [28]–[31]. Biofilms were formed at 2 different inocula (0.5x10^7^ or 0.5x10^4^ CFU). Bacteria from broth cultures were harvested by centrifugation (5000 rpm, 7 minutes), washed in PBS and adjusted to 1x10^7^ or 1x10^4^ CFU/mL in TSBYEHM supplemented with 2% v/v sterile-filtered human saliva (TSBYEHM+S) (ethics approval South Central Oxford C Research Ethics, reference 08/H0606/87+5). To initiate the multispecies biofilm, *S. mitis* (0.5 mL, 0.5x10^7^ or 0.5x10^4^ CFU) was added to SLA Ti, CHX-HMP-100 or CHX-HMP-200 coupons (6 technical replicates) and incubated under anaerobic conditions for 24 hours. Spent medium was removed, the coupon gently washed with 0.5 mL PBS and then transferred to a clean well of a 24-well plate. This was followed by addition of *F. nucleatum* (0.5 mL, 0.5x10^7^ or 0.5x10^4^ CFU). The plate was incubated for a further 24 hours under anaerobic conditions before removal of spent medium, washing of the coupon, transfer to another clean 24-well plate and addition of *A. actinomycetemcomitans, P. gingivalis* and *P. nigrescens* (0.5 mL, 0.5x10^7^ or 0.5x10^4^ CFU of each species). Coupons were incubated anaerobically for a further 24 hours, resulting in a biofilm grown over 72 hours containing five species. A modification to the biofilm formation model was later introduced to clarify the mechanism by which the CHX-HMP coatings affected biomass formation. This consisted of addition of *S. mitis* at time 0 ***and*** at 24 hours (same CFU), followed by inoculation of *F. nucleatum* at 48 hours and *A. actinomycetemcomitans, P. gingivalis* and *P. nigrescens* at 72 hours, as described previously, to give a biofilm grown over 96 hours.

## Supplemental data 4: Methods to assess antibiofilm efficacy of CHX-HMP coatings

The antibiofilm efficacy of the CHX-HMP-100 and CHX-HMP-200 coatings compared to SLA Ti (positive control) was assessed using biomass assays. Biomass on each substrate was determined at 24-hour intervals after removal of medium by staining with 0.5 mL of 0.1% safranin for 10 minutes. Excess stain was removed by washing in PBS and then quantified following release with 1 mL 10% acetic acid and measurement of absorbance at 490 nm. Coupons of each coating type incubated with medium only served as negative controls.

Characterisation of the multispecies biofilm composition was by qPCR was performed after 72 hours incubation. Multispecies biofilms were pooled from 12 specimens per coating type using a cell scraper into a clean 24-well plate containing 1 mL prewarmed PBS. Biomass was harvested by centrifugation (5000 rpm, 7 minutes), and the resultant pellet resuspended in 200 µL PCR dH_2_O. Genomic DNA (gDNA) was then extracted using a MasterPure™ Gram Positive DNA purification kit (Lucigen, Wisconsin, USA) according to the method of Millhouse *et al.* [27].

Primers used for qPCR against the 16S rRNA gene of each target species are shown in supplementary table 1. qPCR reactions (20 µl) comprised 25 pmol each of forward and reverse primer, 2 µl biofilm gDNA template and 10 µl iQ™ SYBR® Green Supermix (Biorad, Watford, UK). All samples were run in duplicate using a CFX Connect™ qPCR machine (BioRad) and the data analysed using CFX Maestro software (BioRad). DNA quantities were determined based on standard curves that had been generated for each test bacterium using gDNA extracted from planktonic suspensions.

Supplementary table 1. Primers used for qPCR.

| **Target bacterium** | **Forward Primer** | **Reverse Primer** | **Source** |
| --- | --- | --- | --- |
| ***A. actinomycetemcomitans*** | GAA CCT TAC CTA CTC TTG ACA TCC GAA | TGC AGC ACC TGT CTC AAA GC | [27], [63] |
| ***P. gingivalis*** | GCG CTC AAC GTT CAG CC | CAC GAA TTC CGC CTG C | [64] |
| ***P. nigrescens*** | CCG TTG AAA GAC GGC CTA A | CCC ATC CCT TAC CGG AA | [65] |
| ***F. nucleatum*** | GGA TTT ATT GGG CGT AAA GC | GGC ATT CCT ACA AAT ATC TAC GAA | [27], [66] |
| ***S. mitis*** | CGA TAC ATA GCC GAC CTG AG | CCA TTG CCG AAG ATT CC | [27] |

## Supplemental data 5: Cytocompatibility testing methods

### Cell culture

Human mesenchymal stromal cells (hMSCs) (Promocell, Germany) were cultured in Dulbecco’s Modified Eagle Medium (DMEM) supplemented with 10% (v/v) foetal bovine serum, 200 mM L-glutamine (Invitrogen, Thermofisher), 100 mM sodium pyruvate, 1% MEM with addition of non-essential amino acids (Gibco, Thermofisher) and antibiotics (6.74 U/mL penicillin-streptomycin, 0.2 µg/mL fungizone) at 37°C in a 5% CO_2_ humidified atmosphere. hMSC monolayers were grown in 75 cm^3^ flasks (Corning Incorporated, Wiesbaden, Germany), washed in HEPES saline buffer (4-(2-hydroxyethyl)-1-piperazineethanesulfonic acid with 0.01 M saline solution) and recovered using trypsin/versene (20 mL versene/0.5 mL trypsin (Sigma)). Media were refreshed twice weekly and cell growth was monitored using a light microscope. Cell density was maintained at approximately 70% by passage. Cells were collected by centrifugation at 1400 rpm for 5 minutes and resuspended in fresh growth medium. hMSCs were enumerated using a haemocytometer and seeded at 10,000 cells/surface. Incubation time was 1, 7, 14, 21 or 28 days according to experiment type, with media replaced twice per week.

### SEM imaging

After 7 days’ growth, medium was removed, specimens washed in PBS and immersed in fixative solution containing 1.5% glutaraldehyde with 0.1 M sodium cacodylate for 1 hour at 4°C. Sodium cacodylate was removed and 1 mL 1% osmium tetroxide in 0.1 M sodium cacodylate added for 1 hour at room temperature. This was followed by three 10-minute washes in DIW and then addition of aqueous 0.5% uranyl acetate for 1 hour in the dark at room temperature. Dehydration was performed using ethanol sequentially at 30%, 50%, 70%, and 90% concentrations for 10 minutes each, followed by absolute ethanol four times for 5 minutes, and the same with dried absolute ethanol. The final drying stage was performed with hexamethyldisilazane for 10 minutes, before samples were placed in a desiccator overnight. Samples were mounted on SEM stubs and sputter coated with gold/palladium alloy (10-20 nm thick) using a Quorum Q105T ES sputter coater (Quorum Technologies Ltd, Laughton, UK). Samples were imaged on a JEOL6400 SEM running at 6-10 kV and images captured using Olympus Scandium software.

### Immunostaining

After 7 days (vinculin and tubulin) or 21 days (osteopontin and osteocalcin) incubation, medium was removed and specimens (in triplicate) fixed in 10% formaldehyde in PBS with 2% sucrose at 37°C for 15 minutes. Substrates were washed in PBS, transferred to new 24-well plates, permeabilisation buffer was added (10.3 g sucrose, 0.292 g sodium chloride, 0.06 g magnesium chloride hexahydrate, 0.476 g HEPES in 100 mL PBS) and plates incubated at 4°C for 5 minutes. The buffer was removed and PBS/1% BSA added and plates incubated for a further 5 minutes at 37°C.

Vinculin, tubulin, osteopontin or osteocalcin primary antibody solutions with phalloidin (rhodamine) were prepared immediately prior to use. Phalloidin was diluted 1:500 in PBS/1% BSA, followed by addition of the relevant antibody (all monoclonal murine) at 1:100 dilution from stock. Antibodies against vinculin and tubulin were supplied by Sigma (V9264 and T0198), and against osteopontin and osteocalcin from Santa Cruz Biotechnology Inc. (sc-21742 and sc-73464). Primary antibody/phalloidin solutions were added (250 µL per well) and plates incubated for 1 hour at 37°C, wrapped in foil. Secondary antibody (biotinylated anti-mouse; Vector Laboratories Ltd, Peterborough, UK) was prepared immediately prior to use by 1:100 dilution in PBS/1% BSA solution. After removal of the primary antibody/phalloidin solution, three 5-minute washes were carried out in PBS/0.5% Tween with gentle shaking. Secondary antibody in PBS/1% BSA was added (250 µl per well) and plates incubated for 1 hour at 37°C, wrapped in foil. After removal of the secondary antibody solution, three 5-minute washes were carried out in PBS/0.5% Tween-20 with gentle shaking. Fluorescein streptavidin was diluted 1:100 in PBS/1% BSA, 250 µL added per well and plates incubated at 4°C for 30 minutes. After removal of this solution, three 5-minute washes were carried out in PBS/0.5% Tween with gentle shaking. Vectashield mounting medium (Vector) containing DAPI to stain cell nuclei was used to mount specimens on a slide. These were stored in the dark at 4°C until imaging under fluorescence with a Zeiss Axiovert 200 M microscope (QCapturePlus software).

### Phosphate staining

After 28 days’ growth, specimens (in duplicate) were stained for phosphate using a modification of the von Kossa method [67]. Medium was removed and specimens washed in 1 mL PBS. Sufficient 5% AgNO_3_ solution was added to cover specimens, which were then exposed to a UV lamp 20 cm distance for 15 minutes. Specimens were rinsed in 1 mL DIW then immersed in 5% sodium thiosulphate for 5 minutes, followed by a 5-minute rinse under tepid running tap water. After a 1 mL rinse with DIW, specimens were counterstained for 5 minutes with nuclear fast red solution (0.1% nuclear fast red, 5% aluminium sulphate) and then final rinses performed in 1 mL DIW and 1 mL 70% ethanol. Specimens were imaged under a light microscope, with silver visible as black deposits.

### Metabolomic analysis

At the end of the growth period assessed (7 or 14 days), spent medium was removed from 4 substrates per coating type. Surfaces were incubated in 400 µl of a chloroform:methanol:DIW solution (1:3:1 ratio) at 4°C for 1 hour with 600 rpm shaking. Liquid was removed and centrifuged for 3 minutes at 10,000 rpm (4°C) to pellet any cell debris. Supernatants were transferred to Eppendorf tubes and frozen individually for each sample at -80°C until analysis. One ‘pooled sample’ comprising 15 µl taken from each extract was also frozen, to produce a reference sample of all metabolites present. An additional ‘CHX control’ coating was incorporated into these assays comprising cells incubated on SLA Ti in medium containing 0.025% chlorhexidine digluconate.

Metabolomic analysis was undertaken at the University of Glasgow Polyomics facility, using liquid chromatography followed by Thermo Extractive orbitrap mass spectrometry (LC-MS). Data processing was carried out using Ingenuity Pathway Analysis software (Qiagen) and Metaboanalyst online software (<https://www.metaboanalyst.ca/>).

## Supplemental data 6: CHX_(aq)_ elution into STF over 14 days

Elution of CHX_(aq)_ from CHX-HMP coated substrates into STF over 14 days is shown in supplemental Figure 1, alongside total available CHX_(aq)_ release into 2 M HCl for comparison. No further significant release was seen after day 1.


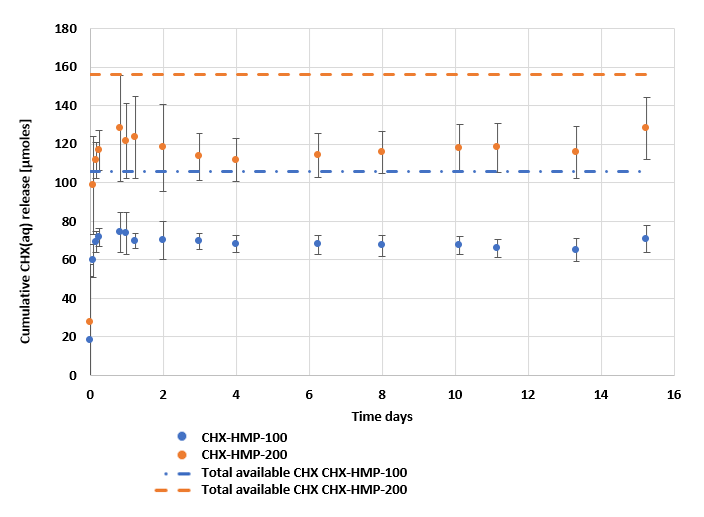


Supplemental Figure 1. Mean cumulative CHX_(aq)_ release per metre squared of evaporatively coated SLA. Elution into STF at room temperature. Error bars show standard deviation. Dotted lines represent pooled mean total available CHX on each coating type as determined using elution into 2 M HCl (10 specimens per coating type).

## Supplemental data 7: statistical analysis of biofilm growth on uncoated and CHX-HMP coated SLA Ti

Supplemental Table 2. Biomass of biofilms grown on uncoated and CHX-HMP coated SLA Ti: 72 hour growth period

| **Inoculum** | **Timepoint** | **Coating** | **Comparison coating** | **P value (1 way Anova and Sidak post hoc, n=3)** |
| --- | --- | --- | --- | --- |
| 0.5x10^4^ CFU/mL | 24 hours | SLA Ti | CHX-HMP-100 | 0.0001 |
|  |  |  | CHX-HMP-200 | 0.0001 |
|  | 48 hours | SLA Ti | CHX-HMP-100 | 0.0001 |
|  |  |  | CHX-HMP-200 | 0.0001 |
|  | 72 hours | SLA Ti | CHX-HMP-100 | 0.0220 |
|  |  |  | CHX-HMP-200 | 0.0010 |
| 0.5x10^7^ CFU/mL | 24 hours | SLA Ti | CHX-HMP-100 | 0.0001 |
|  |  |  | CHX-HMP-200 | 0.0001 |
|  | 48 hours | SLA Ti | CHX-HMP-100 | 0.0001 |
|  |  |  | CHX-HMP-200 | 0.0001 |
|  | 72 hours | SLA Ti | CHX-HMP-100 | 0.0110 |
|  |  |  | CHX-HMP-200 | 0.0600 |

Supplemental Table 3. Biomass of biofilms grown on uncoated and CHX-HMP coated SLA Ti: 96 hour growth period

| **Inoculum** | **Timepoint** | **Coating** | **Comparison coating** | **P value (1 way Anova and Sidak post hoc, n=3)** |
| --- | --- | --- | --- | --- |
| 0.5x10^4^ CFU/mL | 24 hours | SLA Ti | CHX-HMP-100 | 0.0030 |
|  |  |  | CHX-HMP-200 | 0.0030 |
|  | 48 hours | SLA Ti | CHX-HMP-100 | 0.0001 |
|  |  |  | CHX-HMP-200 | 0.0001 |
|  | 72 hours | SLA Ti | CHX-HMP-100 | 0.7170 |
|  |  |  | CHX-HMP-200 | 0.9910 |
|  | 96 hours | SLA Ti | CHX-HMP-100 | 0.9980 |
|  |  |  | CHX-HMP-200 | 1.000 |
| 0.5x10^7^ CFU/mL | 24 hours | SLA Ti | CHX-HMP-100 | 0.1300 |
|  |  |  | CHX-HMP-200 | 0.1340 |
|  | 48 hours | SLA Ti | CHX-HMP-100 | 0.6920 |
|  |  |  | CHX-HMP-200 | 0.5850 |
|  | 72 hours | SLA Ti | CHX-HMP-100 | 1.0000 |
|  |  |  | CHX-HMP-200 | 1.0000 |
|  | 96 hours | SLA Ti | CHX-HMP-100 | 1.0000 |
|  |  |  | CHX-HMP-200 | 1.0000 |

## Supplemental data 8: MIC and MBC to CHXDG_(aq)_

Minimum inhibitory and minimum bactericidal concentrations of CHXDG_(aq)_ determined for each test bacterium, along with comparison to other studies where available, are shown in supplemental Table 4.

Supplemental Table 4. Median MIC and MBC values for bacterial species to CHXDG_(aq)_ with MIC:MBC ratio

|  | ***S. mitis*** | ***F. nucleatum*** | ***A. actinomycetemcomitans*** | ***P. gingivalis*** | ***P. nigrescens*** |
| --- | --- | --- | --- | --- | --- |
| **Experimental MIC % CHXDG_(aq)_**  (µM) | 0.000625  (7.0) | 0.000625  (7.0) | 0.000156  (1.7) | 0.000234  (2.6) | 0.000078  (0.87) |
| **Reported value MIC %**  (µM) | 0.000780  (8.7)  [68;  *S. oralis*] | 0.000195  (2.2)  [68] | 0.000097  (1.1)  [69] | - | 0.000390  (4.3)  [70] |
| **Experimental MBC % CHXDG_(aq)_**  (µM) | 0.002500  (27.8) | 0.001875  (20.9) | 0.000156  (1.7) | 0.003125  (34.8) | 0.003125  (34.8) |
| **Reported value MBC %**  (µM) | 0.000780  (8.7)  [68;  *S. oralis*] | 0.001300  (14.5)  [68] | - | - | 0.000650  (7.2)  [70] |
| **Experimental MIC:MBC ratio** | 1:4 | 1:3 | 1:2 | 1:1.3 | 1:4 |
| **Reported MIC:MBC ratio** | 1:1 | 1:6.6 | - | - | 1:1.6 |

Values reported in other studies, where available, are given alongside for comparison (grey rows). Equivalent concentrations in µM are given in italics in brackets. n=4-6.

## Supplemental data 9: Z-scores generated from metabolomic analysis

Z-scores generated from metabolomic analysis in IPA for relevant cell metabolic functions in hMSCs grown on control and test surfaces are shown in supplemental Table 5. Categories, annotations and z-scores were determined in IPA analysis software. All 7-day test surface scores are relative to 7-day SLA Ti control, as are the 14-day SLA Ti surface scores. All 14-day test surface scores are relative to 14-day SLA Ti control. Only activation scores of greater than 2 (highlighted in orange), or deactivation scores of less than -2 (highlighted in blue) were considered statistically significant in this analysis. For simplicity in presentation, results outside of this range are not presented in the table. Responses are grouped by category.

Supplemental Table 5. Activation and deactivation z-scores for all surfaces

| **Category** | **Disease or Function** | **7 days incubation** | | |  | **14 days incubation** | | | |
| --- | --- | --- | --- | --- | --- | --- | --- | --- | --- |
|  |  | **CHX-HMP-100** | **CHX-HMP-200** | **SLA Ti+0.025% CHXDG_(aq)_** |  | **CHX-HMP-100** | **CHX-HMP-200** | **SLA Ti+0.025% CHXDG_(aq)_** | **SLA Ti** |
|  |  |  |  |  |  |  |  |  |  |
| **Amino Acid Metabolism, Molecular Transport, Small Molecule Biochemistry** | Efflux of L-amino acid |  |  | 2.772 |  |  |  | -2.588 | 2.588 |
|  | Efflux of neutral amino acid |  |  | 2.588 |  | -2.39 | -2.39 | -2.39 | 2.39 |
|  | Efflux of L-alanine |  |  | 2.449 |  | -2.236 | -2.236 | -2.236 | 2.236 |
|  | Uptake of L-proline |  |  | -2.449 |  | 2.449 | 2.449 | 2.449 | -2.449 |
|  | Uptake of L-amino acid |  |  | -3.695 |  | 2.969 | 2.969 | 2.969 | -2.821 |
|  | Uptake of L-alanine |  |  | -3.162 |  | 3 | 3 | 3 | -3 |
|  | Uptake of amino acids |  |  | -3.9 |  | 3.395 | 3.395 | 3.117 | -3.268 |
|  | Uptake of glutamine family amino acid |  |  | -2.828 |  |  |  |  |  |
|  | Transport of amino acids |  |  | -2.479 |  | 2.06 | 2.06 |  |  |
|  | Metabolism of amino acids |  |  |  |  | -2.425 | -2.425 | -2.425 | 2.213 |
|  | Synthesis of amino acids |  |  |  |  | -2.213 | -2.213 | -2.213 |  |
| **Carbohydrate Metabolism, Cellular Function and Maintenance, Molecular Transport, Small Molecule Biochemistry, Energy Production** | Gluconeogenesis |  |  | 2.56 |  |  |  |  | 2.741 |
|  | Transport of D-glucose |  |  | -2.795 |  |  | 2.107 |  | -2.956 |
|  | Oxidation of monosaccharide | -2.352 | -2.131 | -2.741 |  |  |  |  | -2.917 |
|  | Oxidation of glucose-6-phosphate | -2.236 | -2 | -2.449 |  |  |  |  | -2.646 |
|  | Transport of monosaccharide |  |  | -2.903 |  |  | 2.243 |  | -3.058 |
|  | Transport of carbohydrate |  |  | -2.578 |  |  |  |  |  |
| **Cell Cycle** | Entry into S phase |  |  | 2.236 |  |  | -2.828 | -2.121 |  |
| **Cell Death** | Apoptosis | 2.304 | 2.618 |  |  |  |  | 2.082 |  |
|  | Necrosis | 2.116 | 2.258 |  |  |  |  |  |  |
|  | Cell death | 2.078 | 2.562 |  |  |  |  |  |  |
| **Cell Death and Survival, DNA Replication, Recombination, and Repair** | Fragmentation of DNA |  | 2.166 | 2.587 |  |  | -2.107 |  | 2.762 |
|  | Condensation of chromatin |  |  |  |  |  |  |  | 2 |
| **Cell Morphology** | Permeabilization of mitochondria |  |  |  |  |  |  | 2.412 |  |
| **Cell Morphology, Cellular Function and Maintenance** | Transmembrane potential of mitochondria | -2.052 |  |  |  |  |  |  |  |
| **Cell Signalling, Molecular Transport, Small Molecule Biochemistry, Vitamin and Mineral Metabolism** | Release of Ca^2+^ |  |  |  |  |  |  | -2.247 | 2.188 |
|  | Mobilization of Ca^2+^ |  |  | 3.092 |  | -2.541 | -2.489 |  | 3.789 |
|  | Quantity of Ca^2+^ | 3.364 | 2.723 | 3.05 |  |  | -2.089 |  | 3.437 |
|  | Concentration of Ca^2+^ |  |  |  |  |  | -2.604 |  | 2.762 |
| **Cell-To-Cell Signalling and Interaction, Cellular Assembly and Organization, Growth and Proliferation** | Binding of plasma membrane |  |  |  |  | 2.414 | 2.414 |  |  |
|  | Stimulation of cells |  |  | 3.226 |  | -2.71 | -2.549 |  | 3.368 |
|  | Binding of cellular membrane |  |  |  |  | 2.596 | 2.596 | 2.053 |  |
| **Cellular Compromise** | Permeability transition of mitochondria |  |  |  |  | 2.595 |  |  |  |
|  | Permeability transition |  |  |  |  | 2.208 |  |  |  |
|  | Damage of mitochondria |  |  |  |  | 2.177 |  | 2.177 |  |
|  | Dysfunction of mitochondria |  | 2.183 | 2.243 |  |  |  |  |  |
| **Cellular Function and Maintenance** | Respiration of mitochondria |  | 2.213 | 2.415 |  | -2.396 | -2.768 |  | 2.935 |
| **Cellular Growth and Proliferation** | Inhibition of cells |  |  |  |  |  |  |  | 2.689 |
| **DNA Replication, Recombination, and Repair, Nucleic Acid Metabolism, Small Molecule Biochemistry** | Degradation of DNA |  |  |  |  |  | -2.117 |  |  |
|  | Metabolism of DNA |  | 2.383 |  |  |  |  |  |  |
|  | Incorporation of thymidine |  |  |  |  | -2.154 |  |  |  |
| **Drug Metabolism, Molecular Transport, Small Molecule Biochemistry** | Concentration of glutathione |  |  |  |  |  | -2.221 | -2.221 | 2.388 |
| **Energy Production, Molecular Transport, Nucleic Acid Metabolism, Small Molecule Biochemistry** | Consumption of oxygen |  |  |  |  |  | -2.066 |  | 2.383 |
|  | Oxidation of fatty acid |  |  |  |  |  |  |  | 2.085 |
|  | Release of ATP |  |  |  |  | -2.19 | -2.19 |  | 2.19 |
|  | Concentration of ATP |  |  |  |  |  | -2.349 |  |  |
| **Free Radical Scavenging, Small Molecule Biochemistry, Production of Reactive Oxygen Species (ROS)** | Synthesis of nitric oxide |  |  |  |  |  | -2.771 | -2.083 |  |
|  | Release of nitric oxide |  |  |  |  |  |  |  | 2.183 |
|  | Synthesis of ROS | 2.792 | 3.329 | 2.658 |  |  |  | 2.27 |  |
|  | Generation of ROS | 2.274 | 2.222 |  |  |  |  |  |  |
|  | Production of ROS |  | 2.469 | 2.205 |  |  | -2.045 |  |  |
|  | Generation of superoxide |  | 2.433 | 2.449 |  |  |  |  |  |
|  | Production of superoxide |  |  | 2.018 |  |  |  |  |  |
|  | Metabolism of ROS |  |  | 2.512 |  |  |  | 2.265 |  |
|  | Production of lipid peroxide |  |  |  |  |  |  | 2.236 | -2 |
|  | Production of hydrogen peroxide |  |  | 2.033 |  |  | -2.263 |  | 2.074 |
|  | Metabolism of hydrogen peroxide |  |  |  |  |  | -2.09 |  |  |
|  | Biosynthesis of hydrogen peroxide |  |  |  |  |  | -2.136 |  |  |
| **Lipid Metabolism, Molecular Transport, Small Molecule Biochemistry** | Accumulation of acylglycerol |  |  |  |  |  |  |  | 2.361 |
|  | Accumulation of lipid |  |  |  |  |  |  |  | 2.051 |
|  | Uptake of lipid |  |  | -2.147 |  |  |  |  |  |
|  | Quantity of steroid |  |  |  |  | 2.978 |  | 2.097 |  |
|  | Concentration of sterol |  |  |  |  | 2.368 |  |  |  |
|  | Flux of lipid |  |  |  |  |  | -2.01 | -2.33 |  |
|  | Transport of lipid |  |  |  |  |  | -2.069 |  |  |
|  | Hydrolysis of lipid |  |  |  |  |  |  |  | 2.158 |
| **Molecular Transport** | Quantity of metal ion | 3.505 | 2.742 | 3.081 |  |  | -2.134 |  | 3.464 |
|  | Quantity of metal |  |  | 2.877 |  |  |  |  | 3.285 |
|  | Export of molecule |  |  | 2.6 |  | -2.874 | -3.008 | -2.335 | 2.855 |
| **Nucleic Acid Metabolism, Small Molecule Biochemistry** | Synthesis of purine nucleotide |  |  |  |  |  | -2.355 |  | 2.355 |
|  | Synthesis of nucleotide |  |  |  |  |  | -2.061 |  | 2.342 |
|  | Biosynthesis of nucleoside triphosphate |  |  |  |  |  | -2.155 |  | 2.155 |
|  | Biosynthesis of purine ribonucleotide |  |  |  |  |  | -2.132 |  | 2.132 |
| **Organismal Development** | Growth of organism |  |  | 2.169 |  | -3.453 | -2.899 | -3.284 | 2.832 |
| **Protein Synthesis and Trafficking** | Metabolism of protein |  |  | 2.222 |  |  | -2.432 |  | 2.117 |
|  | Synthesis of protein |  |  | 2.037 |  | -2.567 | -2.145 | -2.084 |  |
|  | Interaction of protein |  | 2.216 |  |  |  |  |  |  |

## Supplemental data 10: Networks formed from metabolomic analysis

Apoptosis and necrosis networks produced from metabolomic data are shown in supplemental Figures 3 - 6, with the key to these images shown in supplemental Figure 2. All networks are predicted and produced from 4 substrates per coating type in comparison to hMSCs grown on SLA Ti. For both metabolites and processes, more intense colours indicate a more confident result, paler colours indicate a less confident result.


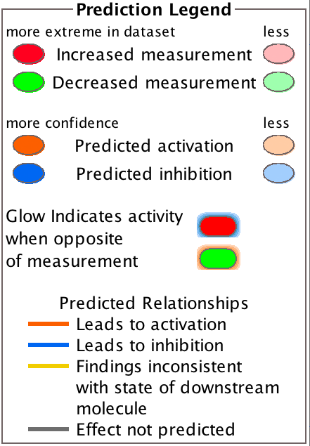


Supplemental Figure 2. Key for metabolomic networks. More intense colours indicate a more confident result, paler colours indicate a less confident result.


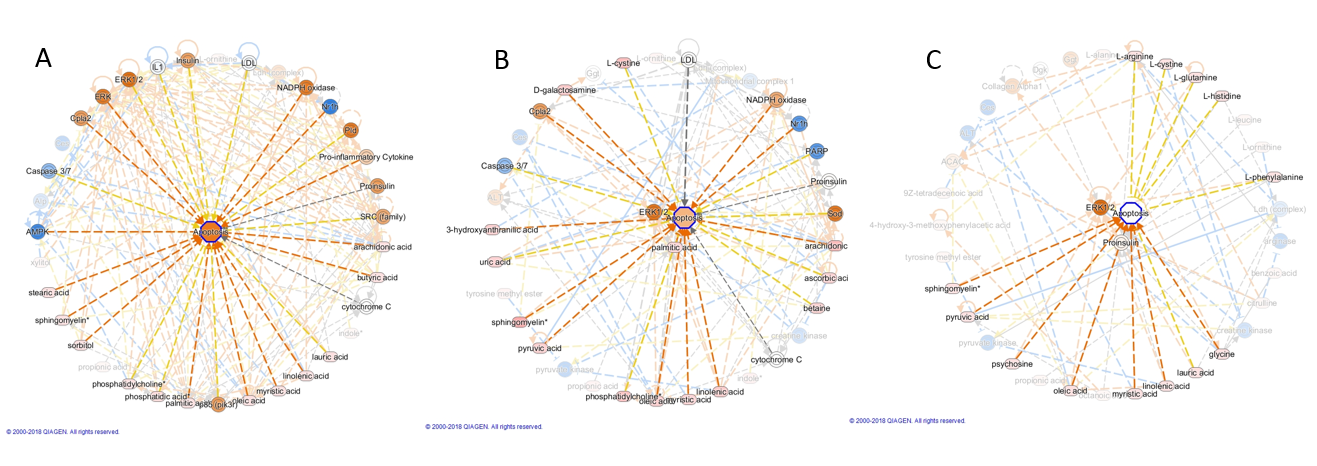


Supplemental Figure 3. Apoptosis networks for test surfaces at 7 days (compared to SLA Ti). A – CHX-HMP-100, B - CHX-HMP-200, C - SLA Ti +0.025% CHXDG_(aq)_. On both CHX-HMP coated surfaces, apoptosis was activated compared to SLA Ti, and mediated in part via the ERK1/2 pathway. On the SLA Ti +0.025% CHXDG_(aq)_ substrate, there was lack of activation of apoptosis, which was unexpected. Increased measurements of a number of common molecules were seen across all 3 surfaces, whilst increased presence of L-amino acids was seen only from cells incubated with 0.025% CHXDG_(aq)_ (as indicated by yellow dashed lines in C) (n=1).


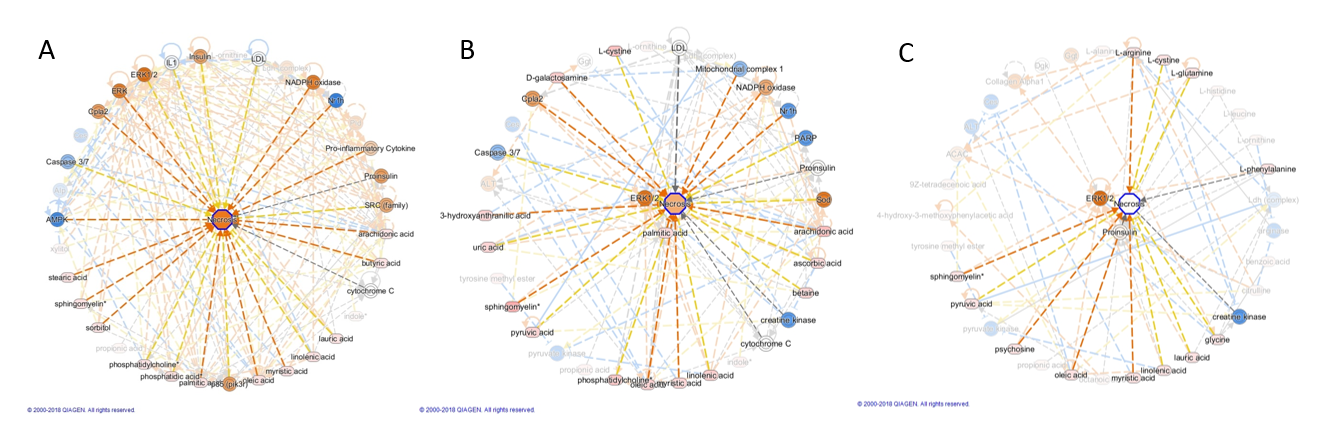


Supplemental Figure 4. Necrosis networks for test surfaces at 7 days (compared to SLA Ti). A – CHX-HMP-100, B - CHX-HMP-200, C - SLA Ti +0.025% CHXDG_(aq)_. On both CHX-HMP coated surfaces, necrosis was activated compared to SLA Ti, whereas on the SLA Ti +0.025% CHXDG_(aq)_, there was lack of necrosis activation (n=1).


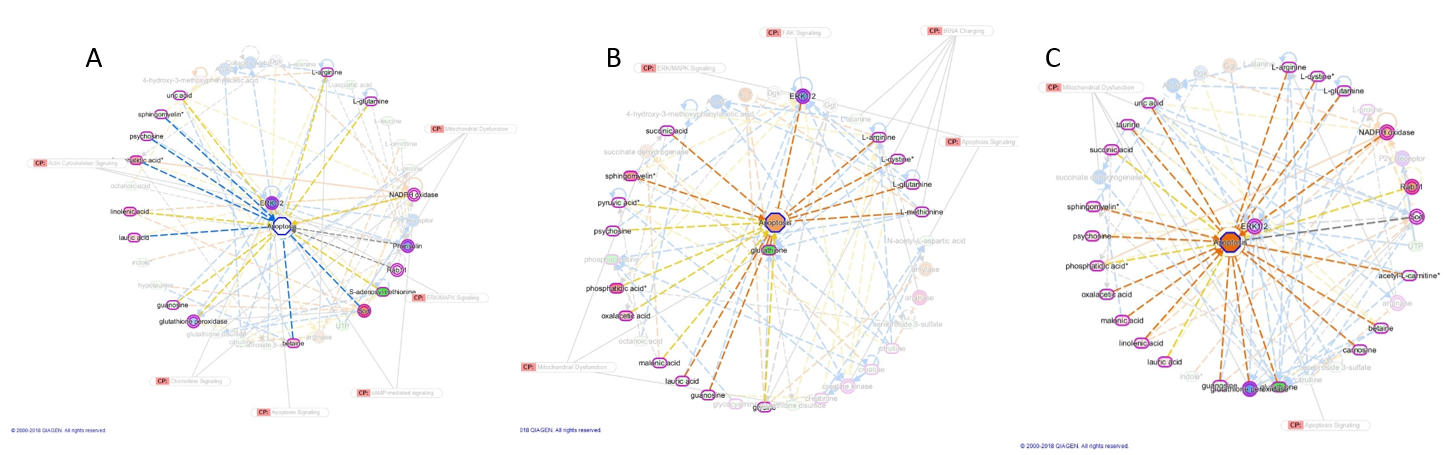


Supplemental Figure 5. Apoptosis networks for test surfaces at 14 days. A – CHX-HMP-100, B - CHX-HMP-200, C - SLA Ti +0.025% CHXDG_(aq)_. Apoptosis was not activated on the lower dose CHX-HMP coating (A), whilst it was activated on the other 2 surfaces (B and C), suggesting a possible dose-response effect of the CHX_(aq)_ (n=1).


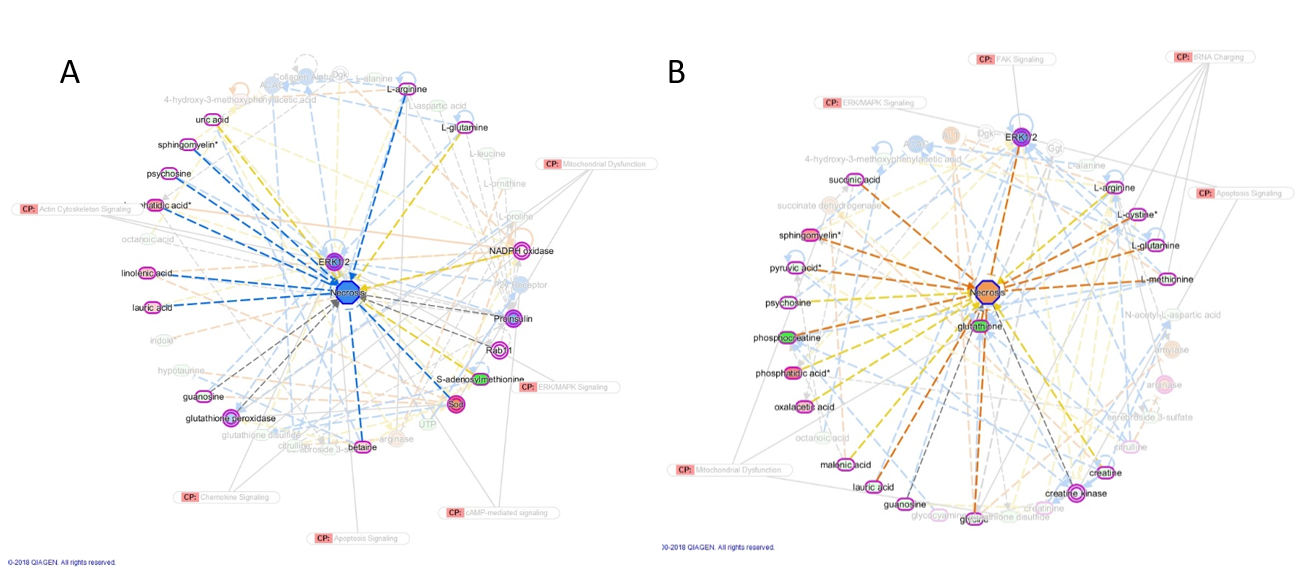


Supplemental Figure 6. Predicted necrosis networks for hMSCs grown on (A) CHX-HMP-100 and (B) CHX-HMP-200 at 14 days compared to SLA Ti (n=1).

References (numbers correspond to references in main text)

[21] Wood NJ, Jenkinson HF, Davis SA, Mann S, O’Sullivan DJ, Barbour ME. Chlorhexidine hexametaphosphate nanoparticles as a novel antimicrobial coating for dental implants. J Mater Sci Mater Med 2015, doi: 10.1007/s10856-015-5532-1.

[27] Millhouse E, Jose A, Sherry L, Lappin DF, Patel N, Middleton AM, Pratten J, Culshaw S, Ramage G. Development of an in vitro periodontal biofilm model for assessing antimicrobial and host modulatory effects of bioactive molecules. BMC Oral Health 2014, doi: 10.1186/1472-6831-14-80.

[28] Subramani K, Jung RE, Molenberg A, Hammerle CHF. Biofilm on dental implants: a review of the literature. Int J Oral Maxillofac Implants, 2009, 24, pp. 616–626.

[29] Leonhardt Å, Renvert S, Dahlén G. Microbial findings at failing implants. Clin Oral Implants Res 1999, doi: 10.1034/j.1600-0501.1999.100501.x.

[30] Mombelli A. Microbiology and antimicrobial therapy of peri-implantitis. Periodontol 2000 2002, doi: 10.1034/j.1600-0757.2002.280107.x.

[31] Charalampakis G, Belibasakis GN. Microbiome of peri-implant infections: Lessons from conventional, molecular and metagenomic analyses. Virulence 2015, doi: 10.4161/21505594.2014.980661.

[63] van der Reijden WA, Brunner J, Bosch-Tijhof CJ, van Trappen S, Rijnsburger MC, de Graaff MPW, van Winkelhoff AJ, Cleenwerck I, de Vos P. Phylogenetic variation of *Aggregatibacter actinomycetemcomitans* serotype e reveals an aberrant distinct evolutionary stable lineage. Infect Genet Evol 2010, doi: 10.1016/j.meegid.2010.07.011.

[64] Boutaga K, Winkelhoff A, Vandenbroucke-Grauls C, Savelkoul P. Comparison of Real-Time PCR and Culture for Detection of *Porphyromonas gingivalis* in Subgingival Plaque Samples. J Clin Microbiol 2003, doi: 10.1128/JCM.41.11.4950-4954.2003.

[65] Kuboniwa M, Amano A, Kimura KR, Sekine S, Kato S, Yamamoto Y, Okahashi N, Iida T, ShizuKuishi S. Quantitative detection of periodontal pathogens using real-time polymerase chain reaction with TaqMan probes. Oral Microbiol Immunol 2004, doi: 10.1111/j.0902-0055.2004.00135.x.

[66] Sánchez M, Marin MJ, Figuero E, Llama-Palacios A, Leon R, Blanc V, Herrera D, Sanz M. Quantitative real-time PCR combined with propidium monoazide for the selective quantification of viable periodontal pathogens in an in vitro subgingival biofilm model. J Periodontal Res 2014, doi: 10.1111/jre.12073.

[67] Mallory F. The Cell - its Component Parts, Constituents and Products,” in *Pathological techniques: A practical manual for workers in pathological histology including directions for the performance of autopsies and for microphotography.*, W. Saunders, Ed. Philadelphia, 1983, pp. 143–144.

[68] McBain AJ, Bartolo RG, Catrenich CE, Charbonneau D, Ledder RG, Gilbert P. Effects of a chlorhexidine gluconate-containing mouthwash on the vitality and antimicrobial susceptibility of in vitro oral bacterial ecosystems. Appl Environ Microbiol 2003, doi: 10.1128/AEM.69.8.4770-4776.2003.

[69] Park JH, Lee JK, Um HS, Chang BS, Lee SY. A periodontitis-associated multispecies model of an oral biofilm. J Periodontal Implant Sci 2014, doi: 10.5051/jpis.2014.44.2.79.

[70] McBain AJ, Ledder RG, Sreenivasan P, Gilbert P. Selection for high-level resistance by chronic triclosan exposure is not universal. J Antimicrob Chemother 2004, doi: 10.1093/jac/dkh168.
